# Annual acknowledgement of manuscript reviewers

**DOI:** 10.1186/2047-9158-3-3

**Published:** 2014-02-11

**Authors:** Shengdi Chen

**Affiliations:** 1 Ruijin Hospital, Shanghai Jiaotong University School of Medicine, Shanghai, China

## Abstract

**Contributing reviewers:**

The editors of *Translational Neurodegeneration* would like to thank all our reviewers who have contributed to the journal in Volume 2 (2013).

## 

William Brooks

Australia

Huaibin Cai

United States of America

Meghan Campbell

United States of America

Piu Chan

China

Honglei Chen

United States of America

Qi Cheng

China

Emilio Ciusani

Italy

Jianqing Ding

China

Yiru Fang

China

Serge Gauthier

Canada

Cheng-Xin Gong

United States of America

Haigang Gu

United States of America

Marwan Hariz

United Kingdom

Maria Trinidad Herrero

Spain

Yue Huang

Australia

Nancy Ip

China

Kurt Jellinger

Austria

Zun-JI Ke

China

Fuzhong Li

United States of America

Jun Liu

China

Weiguo Liu

China

Xuebin Liu

China

Xiao-Guang Luo

China

Guozhao Ma

China

Jianfang Ma

China

Kangmu Ma

United States of America

Jan Marcusson

Sweden

Ian McKeith

United Kingdom

M Maral Mouradian

United States of America

Elizabeth Sajdel-Sulkowska

United States of America

Yuan Shen

China

Hideto Shinno

Japan

Florindo Stella

Brazil

Bomin Sun

China

EK Tan

Slovenia

Beisha Tang

China

Gang Wang

China

Honglin Wang

China

Jian Wang

China

Jianzhi Wang

China

Ning Wang

China

Frances Weaver

United States of America

Howard Worman

United States of America

Ruey-Meei Wu

Taiwan

Zhi-Ying Wu

China

Kun Xia

China

Chen Xianwen

China

Qin Xiao

China

Shifu Xiao

China

Tao Xie

United States of America

Zhongcong Xie

United States of America

Zhen Yan

United States of America

Jingya Zhang

United States of America

Jialin Zheng

United States of America

Jiawei Zhou

China

Xiongwei Zhu

United States of America

